# Macrophages Enhance Migration in Inflammatory Breast Cancer Cells via RhoC GTPase Signaling

**DOI:** 10.1038/srep39190

**Published:** 2016-12-19

**Authors:** Steven G. Allen, Yu-Chih Chen, Julie M. Madden, Chelsea L. Fournier, Megan A. Altemus, Ayse B. Hiziroglu, Yu-Heng Cheng, Zhi Fen Wu, Liwei Bao, Joel A. Yates, Euisik Yoon, Sofia D. Merajver

**Affiliations:** 1 Program in Cellular and Molecular Biology, University of Michigan Medical School, Ann Arbor, MI, 48109, USA; 2 Medical Scientist Training Program, University of Michigan Medical School, Ann Arbor, MI, 48109, USA; 3 Department of Internal Medicine, University of Michigan, Ann Arbor, MI, 48109, USA; 4 Department of Electrical Engineering and Computer Science, University of Michigan, Ann Arbor, MI 48109, USA; 5 University of Michigan Comprehensive Cancer Center, Ann Arbor, MI, 48109, USA; 6 Office for Health Equity and Inclusion, University of Michigan, Ann Arbor, MI, 48109, USA; 7 Program in Cancer Biology, University of Michigan Medical School, Ann Arbor, MI 48109, USA; 8 Department of Biomedical Engineering, University of Michigan, Ann Arbor, MI 48109, USA

## Abstract

Inflammatory breast cancer (IBC) is the most lethal form of breast cancer. All IBC patients have lymph node involvement and one-third of patients already have distant metastasis at diagnosis. This propensity for metastasis is a hallmark of IBC distinguishing it from less lethal non-inflammatory breast cancers (nIBC). Genetic profiling studies have been conducted to differentiate IBC from nIBC, but no IBC cancer-cell-specific gene signature has been identified. We hypothesized that a tumor-extrinsic factor, notably tumor-associated macrophages, promotes and contributes to IBC’s extreme metastatic phenotype. To this end, we studied the effect of macrophage-conditioned media (MCM) on IBC. We show that two IBC cell lines are hyper-responsive to MCM as compared to normal-like breast and aggressive nIBC cell lines. We further interrogated IBC’s hyper-responsiveness to MCM using a microfluidic migration device, which permits individual cell migration path tracing. We found the MCM “primes” the IBC cells’ cellular machinery to become extremely migratory in response to a chemoattractant. We determined that interleukins −6, −8, and −10 within the MCM are sufficient to stimulate this enhanced IBC migration effect, and that the known metastatic oncogene, RhoC GTPase, is necessary for the enhanced migration response.

Inflammatory breast cancer (IBC) is a rare and very aggressive form of breast cancer with the poorest prognosis^1^,^2^,^3^,^4^. IBC is characterized by a rapid onset (by definition within 6 months) of symptoms comprising breast erythema, edema which may contribute to a pitted appearance of the overlying skin termed peau d’orange, and occasional ulceration^1^,^2^,^3^. A definitive diagnosis of IBC is made in a patient with these clinical symptoms and short timeline coupled with pathologic confirmation of invasive carcinoma^3^. Although IBC has a low incidence (about 2% in the United States^1^,^2^,^4^), it is the most lethal form of breast cancer with a median survival of approximately 4 years compared to >10 years for other non-inflammatory breast cancers (nIBC)^4^,^5^,^6^. A key characteristic of IBC distinguishing it from nIBC is IBC’s propensity for metastasis. Essentially all IBC patients present with lymph node involvement and one-third of patients already have distant metastasis at initial diagnosis^1^,^2^,^5^,^6^. The survival curves for metastatic nIBC and non-metastatic IBC are nearly identical the first five years post diagnosis, further highlighting IBC’s characteristic lethality and rapid metastasis^7^.

Many genetic profiling studies have been conducted to try and discern the specific differences between IBC and nIBC that drive the dramatic disparity in mortality^8^,^9^,^10^,^11^,^12^. However, the overarching conclusion of these studies is that no molecular signature can be considered conclusive to adequately distinguish IBC from nIBC^2^,^8^. The 5 general molecular subtypes of nIBC (luminal A, luminal B, basal-like, HER2-enriched, and normal-like) are also represented in IBC, and IBC patients have a poorer prognosis regardless of the subtype^5^,^6^,^10^,^13^,^14^. A recent study determined that initial findings in differential gene expression between IBC and nIBC were in fact due to a difference in proportion of the 5 subtypes (IBC has fewer Luminal A and greater HER2-enriched cancers); when subtypes were directly compared, all IBC vs. nIBC expression differences disappeared^10^. Yet another study looked at histologic features in nIBC that can predict patient outcomes and found such markers had no predictive effect in IBC, which led the authors to conclude that IBC has a distinct biological behavior^15^. One of the few proteins that is continuously found to be differentially expressed between IBC and nIBC is the Ras homology GTPase RhoC^16^,^17^,^18^,^19^. Rho proteins are involved in the actin cytoskeleton turnover and are important for cell motility and focal adhesion kinetics^20^,^21^,^22^. Rho GTPases also signal to a variety of downstream effectors to influence cell survival and proliferation, as well as to functions that impact cancer progression, such as angiogenesis and matrix degradation^20^,^21^. While it is not a marker exclusively specific for IBC (as it is expressed in some aggressive nIBCs), RhoC has a major role specific to cancer cell migration^23^,^24^. Moreover, RhoC is overexpressed in over 90% of IBC and has been shown to be a driver of IBC metastasis^18^,^19^,^25^,^26^,^27^.

The focus on tumor-intrinsic features such as gene expression and the recent finding of a stromal gene signature associated with IBC have yielded helpful, but mechanistically limited, explanatory power for the particularly motile nature of IBC, which may play an important role in its metastatic potential^28^. We hypothesized that hypersensitivity of IBC to tumor-*extrinsic* factors may help account for the differences in behavior between IBC and nIBC. Owing to the importance of the immune components as cancer cell extrinsic elements in the tumor microenvironment and specifically to the role of macrophages in chemotaxis^29^,^30^,^31^,^32^, we sought to determine if tumor-associated macrophages (TAMs) promote IBC’s extreme metastatic nature. TAMs have been shown to have a wide range of pro-tumor effects including supporting angiogenesis, increasing tumor cell invasion and migration, suppressing antitumor responses, and even promoting metastasis^29^,^30^,^31^,^32^.

In this work, we show that the IBC cell lines SUM190 and SUM149 are hyper-responsive to macrophage-conditioned media (MCM) as compared to the normal-like MCF10A breast cell line and the aggressive nIBC MDA-MB-231 cell line. We further interrogated the enhanced IBC migratory phenotype to MCM using a microfluidic migration device. This device allows for individual cell positional information yielding insights into the specific migration pattern of diverse cell populations. The MCM acted to “prime” the IBC cells’ cellular migration machinery to become hyper-responsive to serum chemoattractant and subsequently extremely migratory. At the molecular level, we determined that interleukins −6, −8, and −10 within the MCM are sufficient to stimulate this enhanced IBC migration. Furthermore, we found that the known metastasis-associated oncogene, RhoC GTPase, is necessary for the enhanced migration response and for the MCM activation of components of the mitogen-activated protein kinase (MAPK) cascade.

## Results

### Macrophage-conditioned media enhances IBC migration

**Figure 1:**
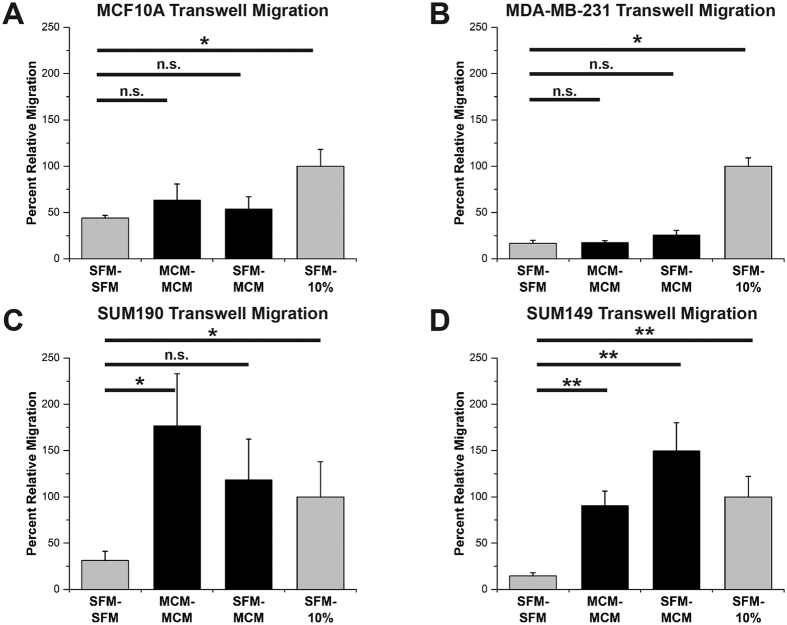
Inflammatory breast cancer cells are hyper-responsive to macrophage conditioned media. Transwell migration assays of (**A**) normal-like MCF10A, (**B**) non-inflammatory MDA-MB-231, (**C**) inflammatory SUM190, and (**D**) inflammatory SUM149 cells. In (**A**) and (**B**), MCF10A and MDA-MB-231 were indifferent to stimulation with macrophage-conditioned media (MCM) migrating similarly to the serum-free media negative control (SFM – SFM). In (**C**) and (**D**), SUM190 and SUM149 IBC cells were hyper-responsive to stimulation with MCM as compared to the SFM – SFM condition. For all, the entire transwell membrane was imaged and the area of migrated cells calculated. All conditions were normalized to the SFM – 10% serum positive control condition as 100% migration. *Denotes p < 0.05, **denotes p < 0.01, Mann-Whitney U test, error bars represent s.e.m.

The normal-like MCF10A, aggressive nIBC MDA-MB-231, and IBC SUM190 and SUM149 cell lines were evaluated for their migration responsiveness to serum and macrophage-conditioned media (MCM) in transwell migration chambers. Representative transwell migration membranes for each cell line and an experimental schematic are shown in Supplementary Fig. S1. MCM was generated from phorbol 12-myristate 13-acetate (PMA) differentiated U937 cells (see Methods)^33^,^34^. MCF10A and MDA-MB-231 cells did not migrate more than the negative control (serum free media in the top and bottom chambers, SFM – SFM) when exposed to MCM as a gradient (SFM–MCM) or no gradient (MCM – MCM) (columns 1, 2, and 3 of Fig. 1A,B). The number of cells migrating when exposed to the positive control, a 10% serum gradient (SFM – 10%), was set as 100% relative migration (column 4 throughout Fig. 1). In contrast, both the IBC cell lines, SUM190 and SUM149, were significantly more motile merely in the presence of MCM without a concentration gradient (column 2 of Fig. 1C,D). Furthermore, SUM149 IBC cells had significantly enhanced migration toward an MCM gradient and, while not significant, an MCM gradient induced a 4-fold increase in migration over SFM negative control in SUM190 IBC cells (column 3 of Fig. 1C,D). Taken together, these results demonstrate an increased migratory responsiveness of the IBC cell lines to factors in the MCM, whereas the normal-like and nIBC cell lines were motility-indifferent to the secreted macrophage factors.

**Figure 2:**
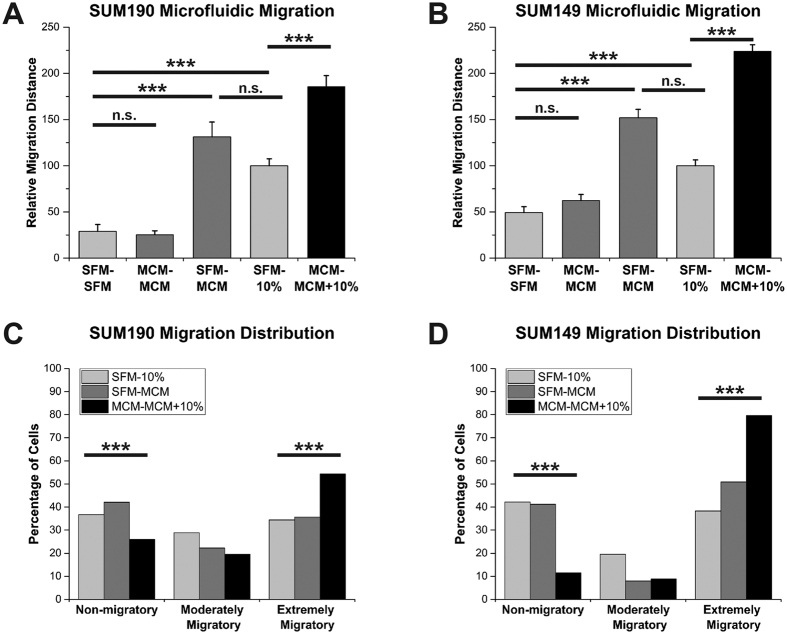
Macrophage conditioned media induces extreme migration in inflammatory breast cancer cells. Microfluidic migration device assays of (**A**) SUM190 and (**B**) SUM149 cells. Both SUM190 and SUM149 had a 2-fold increased migration distance to the MCM – MCM + 10% serum condition (column 5) over SFM – 10% serum positive control (column 4). For (**C**) and (**D**), SUM190 and SUM149 migration distances were parsed into 3 groups: non-migratory were cells with a migration distance less than the SFM – SFM average distance, extremely migratory cells were those with a migration distance greater than the SFM – 10% serum average, and moderately migratory cells had distances between these averages. The percentage of total cells across all experiments for each group is plotted. The MCM – MCM + 10% serum condition (black columns) stimulated non-migratory cells to become extremely migratory cells in SUM190 and SUM149. ***Denotes p < 0.001, Kruskal-Wallis H test (**A,B**), Pearson’s chi-squared test (**C,D**), error bars represent s.e.m.

Given that the IBC cell lines were more responsive to the MCM than the nIBC cell lines, we next sought to accurately quantify the magnitude of this response and to understand if certain subpopulations of the IBC cells were responsible for this behavior. Transwell migration devices are limited in that they reduce a cell’s response to a binary outcome–successful migration or not. As such, they yield no insight into the potential range of motility behaviors between these two extremes, and data from individual cells within a heterogeneous population is lost to bulk analysis. Therefore, in order to facilitate the tracking of individual cell migration paths and quantify single-cell responses to the stimuli, we utilized a microfluidic device with a series of horizontal migration channels that enables the measurement of single-cell positional information throughout the migration experiment (Supplementary Fig. S2)^35^,^36^. In these devices, a passive diffusion concentration gradient can be generated from the left side of the device to the right side by loading the experimental media conditions into the left and right reservoirs (Supplementary Fig. S2B), similar to the top and bottom chambers of a transwell assay respectively. Cells are loaded along the left side of the device and then attracted to migrate toward the right side where there is a higher concentration of soluble factors. Using the microfluidic devices, we discovered that the increase in migration responsiveness was not due to undirected increased chemomotility since the MCM – MCM condition was not different from SFM – SFM condition (columns 1 and 2 of Fig. 2A,B). Instead the increased migration with MCM was in fact due to a statistically significant enhanced capability of both SUM190 and SUM149 IBC cells to chemotax about twice the distance toward the serum gradient in the presence of MCM stimulation (MCM – MCM + 10% serum 2x greater than SFM – 10% serum) (columns 4 and 5 of Fig. 2A,B). This distinction was likely only possible through having migration distance information on a per-cell basis rather than the binary output of a transwell assay. SUM190 and SUM149 cells also respectively migrated 1.3 and 1.5 times further toward an MCM gradient than toward the 10% serum gradient controls demonstrating that the MCM might act as a chemoattractant; however, these increases over the serum control were not statistically significant (columns 3 and 4 of Fig. 2A,B). The design and output of the microfluidic migration devices afforded a closer inspection of the distribution of individual cell migration distances, which revealed two migration subpopulations within the IBC cell lines (Fig. 2C,D). In the SFM – 10% serum and SFM–MCM conditions, a bimodal distribution of approximately equal percentages of cells is apparent comprising of non-migratory cells (defined as those cells migrating less than the SFM – SFM average distance) and extremely migratory cells (defined as those cells migrating further than the SFM – 10% serum average distance) (Fig. 2C,D). These two groups account for about 70–90% of all the IBC cells in the SFM – 10% serum and SFM–MCM conditions. However, the majority of IBC cells in the MCM – MCM + 10% serum condition are stimulated to become extremely migratory cells. With SUM149, the non-migratory cell percentage dropped from 40% of the population to 10% and the extremely migratory percentage statistically significantly increased a proportionate amount from 50% to 80% when comparing the SFM–MCM and MCM – MCM + 10% serum conditions (Fig. 2D). Likewise, a statistically significant trend of a decreasing percentage of non-migratory and a proportionate increasing percentage of extremely migratory cells was also seen with SUM190 (Fig. 2C). The significantly enhanced chemotaxis in the MCM – MCM + 10% serum groups is directly related to the presence of the MCM on the left side of the device stimulating all the cells at the starting location, which prompts the conversion of the non-migratory cell population into extremely migratory cells. In essence, when the moderately migratory condition of a 10% serum gradient was superimposed on the non-migratory MCM – MCM condition, the result is a super migratory response (columns 2, 4, and 5 of Fig. 2A,B; see also Supplementary Fig. S2D). This led us to conclude that it was not necessarily a specific factor in the conditioned media acting as the chemoattractant leading to enhanced migration in the MCM – MCM + 10% serum groups; in fact there was no concentration gradient to the MCM factors and thus no chemotactic signal generated. Instead the presence of the cytokine milieu acted to “prime” the IBC cells’ cellular migration machinery to become hyper-responsive to the superimposed serum gradient, and thus subsequently extremely migratory.

### Interleukins −6, −8, and −10 are sufficient to enhance IBC migration

**Figure 3:**
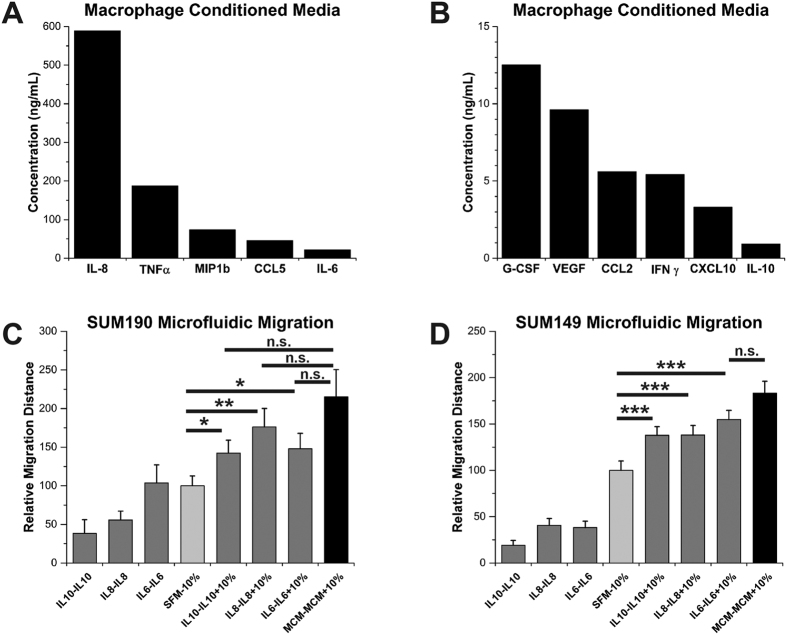
Interleukins −6, −8, and −10 enhance inflammatory breast cancer migration. The concentration of selected cytokines in MCM is plotted in (**A**) and (**B**). In (**C**) and (**D**), microfluidic migration to cytokine conditions is plotted for SUM190 and SUM149 cells, respectively. IL-6, IL-8, and IL-10 were sufficient in isolation (columns 5, 6, and 7) to significantly enhance migration in both cell lines over the SFM – 10% serum control, partially recapitulating the effect seen with the full complement of MCM factors (column 8). *Denotes p < 0.05, **denotes p < 0.01, ***denotes p < 0.001, Mann-Whitney U test, error bars represent s.e.m.

In order to determine what molecular components of the macrophage-conditioned media were promoting the enhanced migration of the IBC cells, we profiled the MCM using a bead-based 27-plex ELISA and then designed a screening protocol to select the components most likely to contribute to the observed migration phenomenon. The concentrations of selected cytokines in the MCM are plotted in Fig. 3 A and B. All 27 cytokine concentrations in MCM and other conditioned media are shown in Supplementary Fig. S3A and B. Cytokines interleukin (IL) −8, tumor necrosis factor α (TNFα), chemokine C-C motif ligand 5 (CCL5), IL-6, vascular endothelial cell growth factor (VEGF), CCL2, and IL-10 were selected for transwell migration screening of enhanced migration based on their high concentrations and likely involvement in nIBC^37^. Cytokines were tested in migration assays at similar concentrations to those measured in the MCM. Of the cytokines screened, IL-6, IL-8, and IL-10 were sufficient to induce a qualitatively enhanced migration response in the transwell assays (data not shown) and therefore the migration response to these factors was further quantitatively assessed in the microfluidic devices. For each cytokine, a no gradient control (equal concentrations of the cytokine in the top and bottom chambers: cytokine – cytokine) and a serum-spiked condition (cytokine – cytokine + 10% serum) mimicking the MCM – MCM + 10% serum condition were performed. In the microfluidic migration assay, all three cytokine – cytokine + 10% serum conditions tested (IL-6, IL-8, and IL-10) significantly enhanced migration over the 10% serum gradient controls across both IBC cell lines, partially recapitulating the MCM – MCM + 10% serum extreme migration effect (Fig. 3C,D). Although the magnitude of the enhanced microfluidic migration effect was tempered, it was robust enough that in SUM149 cells the IL6 – IL6 + 10% serum condition was not significantly different from the MCM – MCM + 10% serum condition, and in SUM190 cells none of the three cytokine – cytokine + 10% serum conditions were significantly different from the MCM – MCM + 10% serum condition (Fig. 3C,D). This supports the importance of these three cytokines and their sufficiency to induce extreme migration in IBC cells. Thus we determined that interleukins −6, −8, and −10 are key cytokines within the MCM stimulating the IBC enhanced migration response.

### RhoC GTPase is necessary for the IBC extreme migration and MCM activation of components of the MAPK cascade

**Figure 4:**
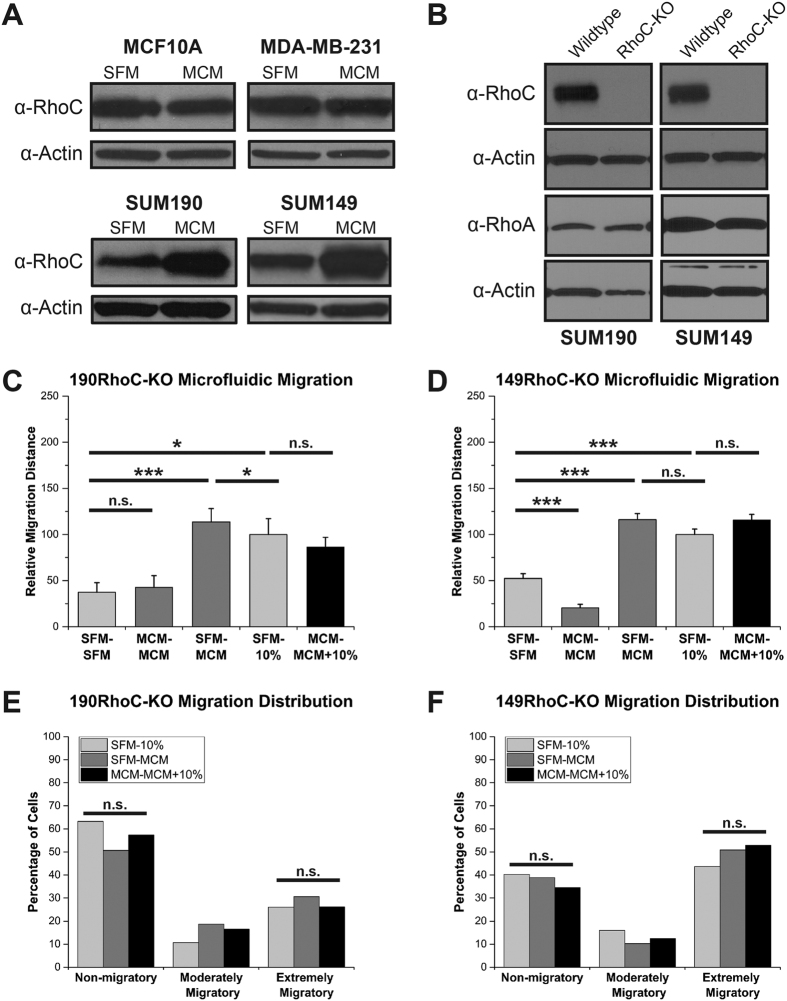
RhoC is necessary for the inflammatory breast cancer migration response to macrophage conditioned media. (**A**) MCM increased the expression of RhoC in SUM190 and SUM149 but not in MCF10A or MDA-MB-231 cells. Immunoblots shown are representative of >3 separate experiments. (**B**) Immunoblotting confirmation of CRISPR knockout of RhoC and not of RhoA. In (**C**) and (**D**), microfluidic migration of the SUM190 and SUM149 CRISPR RhoC knockout cell lines, respectively. RhoC is necessary for the enhanced migration effect as the MCM – MCM + 10% serum migration (column 5) is not different from SFM – 10% serum control (column 4). The migration distribution of 190RhoC-KO (**E**) and 149RhoC-KO (**F**) cells are plotted. RhoC is also necessary for the shift in non-migratory cells to extremely migratory cells. *Denotes p < 0.05, ***denotes p < 0.001, Kruskal-Wallis H test (**C,D**), Pearson’s chi-squared test (**E,F**), error bars represent s.e.m.

Given that RhoC GTPase has been shown to be a key driver of IBC metastasis in *in vivo* models^26^ and it is differentially expressed between IBC and nIBC tumors across studies^16^,^17^,^19^, we hypothesized that RhoC plays a role in the enhanced migration response of IBC to the MCM. In support of this, both IBC cell lines, SUM190 and SUM149, had an increase in RhoC expression upon stimulation with MCM, while there was no change in RhoC expression in either the normal-like MCF10A or the nIBC MDA-MB-231 cells (Fig. 4A). To further test the function of RhoC in this context, we utilized the clustered regularly interspaced short palindromic repeats (CRISPR)–Cas9 system targeting RhoC to knock out the gene in both SUM190 (190RhoC-KO) and SUM149 (149RhoC-KO) cell lines (Fig. 4B). The knockout was specific to RhoC and did not have an effect on the expression of the highly homologous RhoA GTPase (Fig. 4B). As seen in Fig. 4C and D, knocking out RhoC expression had the effect of specifically and completely abolishing the extreme migration of the MCM – MCM + 10% serum condition in both 190RhoC-KO and 149RhoC-KO cells in the microfluidic migration assay (columns 4 and 5 of Fig. 4C,D). Verifying that the CRISPR RhoC knockout effect was specific to the MCM enhanced migration and did not simply abrogate all migration, the 190RhoC-KO and 149RhoC-KO cells were still able to migrate to the 10% serum gradient as robustly as wildtype SUM190 and SUM149 (Supplementary Fig. S4). Additionally, in the RhoC knockout IBC cells, there was no statistically significant increase in the extremely migratory subpopulation when stimulated with the MCM – MCM + 10% serum condition (Fig. 4E,F) demonstrating that without RhoC the IBC cells are unable to be “primed” to become extreme migrators.

**Figure 5:**
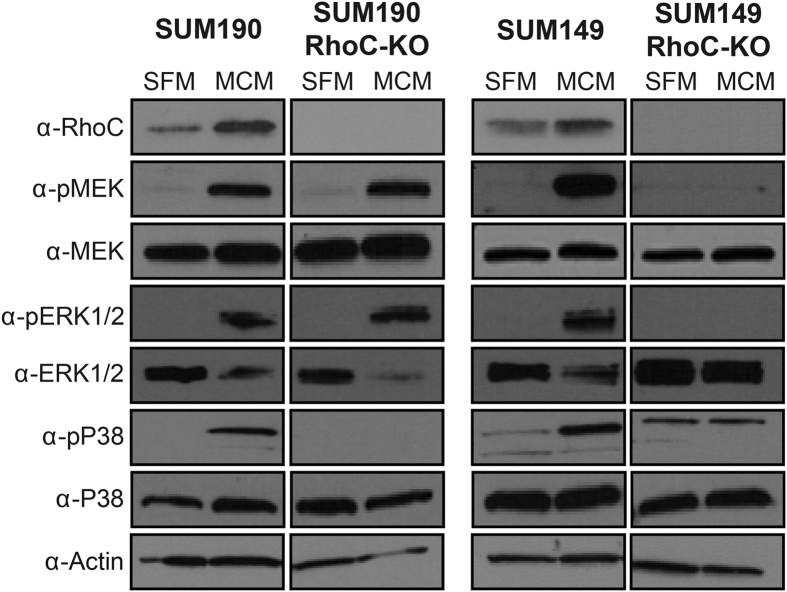
Macrophage conditioned media activates the MAPK cascade in inflammatory breast cancer cells. Immunoblotting for the indicated phospho-proteins and total proteins. RhoC was necessary for the MCM-induced phosphorylation of MEK, ERK1/2, and p38 in SUM149 cells and RhoC was necessary for the MCM-induced phosphorylation of p38 in SUM190 cells. Immunoblots shown are representative of 3 separate experiments.

Furthermore, since the mitogen-activated protein kinase (MAPK) pathway has previously been shown to be important in RhoC signaling, we investigated its role in MCM-induced IBC cell migration^18^. MCM stimulation of wildtype SUM190 and SUM149 induces phosphorylation and activation of MEK, ERK1/2, and p38 (Fig. 5). In 149RhoC-KO cells, stimulation with MCM failed to phosphorylate any of these proteins in the absence of RhoC (Fig. 5). In 190RhoC-KO cells, p38 phosphorylation is completely abrogated while MEK and ERK1/2 remain activated in the absence of RhoC (Fig. 5). We conclude from this series of experiments that RhoC expression is absolutely essential for the MCM-induced enhanced migration of IBC cells as the CRISPR RhoC knockout cell lines lost all evidence of extreme migration in the presence of MCM. Our data also suggests that the MCM plausibly drives this hyper-responsiveness through signaling via components of the MAPK pathway in both IBC cell lines.

## Discussion

Many studies have sought to understand the genetic determinants of the IBC phenotype^8^,^9^,^10^,^15^. However, taken together, this research has demonstrated that broad whole genome expression or mutational studies of the cancer cells themselves do not discern between IBC and nIBC^2^,^10^. This led us to conjecture that factors extrinsic to the cancer cells might explain IBC’s pronounced metastatic propensity; thus, we tested whether tumor-associated macrophages could be contributing to the phenotype through stimulation of RhoC. In accordance with our hypothesis, we postulate that the breast parenchyma in certain individuals–modified by breastfeeding, pregnancy, and body-mass index, which all affect the stromal components^7^ –might provide the proper “soil” for IBC to develop as a metastatic lesion from its inception.

In this study, we found that two IBC cell lines, SUM190 and SUM149, were hyper-responsive to MCM with regard to motility as compared to the normal-like MCF10A and nIBC MDA-MB-231 cell lines. To analyze this behavior further, we designed a microfluidic migration device that enabled the accurate tracking of individual cells to glean the precise magnitude of their response–a measurement that is not possible with the limited qualitative output of traditional transwell assays. We demonstrated that stimulating the IBC cells with MCM doubles their migration to a serum gradient in our microfluidic device. Importantly, our experiments were controlled to provide the definitive information that the MCM itself is not the chemoattractant directly causing the increased migration *per se*, since in the extreme migratory condition there is no gradient to factors in the MCM (analogous to the MCM – MCM condition alone which did not increase migration over the SFM – SFM negative control). Therefore, our data support that components of the macrophage-conditioned media serve to “prime” the IBC cells to become hyper-responsive and extremely migratory when they are further exposed to the directional chemoattractant signal from a serum gradient. When exposed to MCM, IBC cells chemotax twice as far toward a serum gradient than they would without MCM stimulation. The possibility exists that the MCM is inducing a cell-specific autocrine response from the IBC cells which in turn triggers the extreme migration, but the conclusion and net migration result remains the same for both cells lines. Our microfluidic migration devices also allowed us to discern that, within SUM190 and SUM149, there exists a population of cells that are intrinsically extremely migratory toward a chemotactic gradient. Upon stimulation with MCM on both sides of the device (removing a gradient to MCM factors), and superimposing a serum gradient (MCM – MCM + 10% serum), many of the previous would-be non-migratory cells were converted to extremely migratory cells.

Among the MCM factors that could be contributing to this primed-for-hyper-response migration phenotype, we found that interleukins −6, −8, and −10 alone were sufficient to recapitulate the enhanced migration effect. When exposed to a non-gradient condition for these cytokines superimposed with a serum gradient (cytokine – cytokine + 10% serum), both SUM190 and SUM149 had significantly increased microfluidic migration over the serum gradient control alone (SFM – 10% serum). While significant, this increase in migration was more modest as compared to the doubling of migration seen with the total MCM. Thus, it is likely that a mixture of cytokines found in the MCM act in concert to induce the extreme migration effect. However, our finding that these three cytokines–IL-6, IL-8, and IL-10–can directly contribute to IBC’s metastatic phenotype is in agreement with a recent study that showed in a canine model of IBC tissue homogenate levels of IL-6, IL-8, and IL-10 were significantly higher than in canine nIBC^38^. Furthermore, in nIBC, patient serum levels of IL-6, IL-8, and IL-10 all rise with increasing stage supporting their association with invasion and metastasis^37^,^39^. These *in vivo* studies corroborate our finding of the importance of IL-6, IL-8, and IL-10 in IBC migration.

Our results are also in keeping with other recent studies that profiled the importance of macrophages in IBC^40^,^41^,^42^. Cohen *et al*. studied the epithelial-mesenchymal transition in IBC cell lines and found IL-6 to be one essential driver of the transition as measured by qRT-PCR of a standard gene panel^40^. Mohamed *et al*. in another series of experiments investigated the effect of conditioned media from undifferentiated U937 cells on SUM149 and later isolated CD14+ leukocytes directly from the draining blood vessels supplying IBC and nIBC tumors during surgery^41^,^42^. This patient-based study showed that not only do IBC patients have greater staining for CD14+ monocytes in tumor sections, but that IL-8 and IL-10 were especially salient cytokines that were significantly differentially expressed between IBC and nIBC macrophages^41^.

Our work herein demonstrates one specific and robust mechanism that may be active in the processes elicited by the interactions between the macrophage-derived cytokines and the IBC cells suggested by the *in vivo* studies. Rather than acting as a bona fide chemoattractant itself, we propose that the macrophage-conditioned media “primes” the IBC cells by stimulating them into a state ready for a magnified migration analogous to revving the engine of a car in neutral. Then, when a separate chemotactic signal is received (in our experiments the 10% serum gradient), the IBC cells with their engines revved migrate twice as far.

Furthermore, we conclude that RhoC is necessary for the MCM-induced enhanced migration in both SUM190 and SUM149, and our work supports that RhoC plausibly mediates the effect by signaling through components of the MAPK cascade. Using CRISPR RhoC knockouts of the IBC cell lines, the increased migration in the MCM – MCM + 10% serum condition is completely abrogated and the RhoC knockout cell lines migrate no further than they do to SFM – 10% serum. Additionally, without RhoC, the IBC cells are unable to be “primed” by the MCM and do not convert non-migratory cells into extremely migratory cells. These results clearly demonstrate that RhoC is required in IBC for the observed effect. Importantly as a control, the RhoC knockout cells can migrate as well as wildtype IBC cells can toward a serum gradient giving further evidence of the specificity of the RhoC knockout for the MCM-priming and super migration effect. MCM also increases phosphorylation of components of the MAPK pathway in both SUM190 and SUM149 cells. This pathway activation is completely abrogated in SUM149 cells in the absence of RhoC. In 190RhoC-KO cells, p38 signaling is abolished while MEK and ERK1/2 remain activated when treated with MCM. In contrast to SUM149 cells which are triple negative, SUM190 cells have HER-2 overexpression and some components of the MAPK pathway may have a higher basal level of activity thus accounting for the differential signaling between SUM190 and SUM149 RhoC knockout cells^43^. This is further evidence that IBC is a heterogeneous disease at the molecular level as demonstrated by the difference in signaling between SUM190 and SUM149 cells. Yet while the precise patterns of phosphorylation and activation differ between the two cell lines, the two IBC cell lines share the commonality of enhanced migration to macrophage-secreted cytokines through the common signaling node of RhoC.

Thus, our work reveals both a role for the microenvironment in tumor-associated macrophage secreted cytokines and lends further support for RhoC as a potential target for therapeutic intervention aimed at preventing the metastasis of inflammatory breast cancer. Further work could expand on ours and develop specific RhoC GTPase inhibitors as RhoC was shown to be necessary for the IBC super migration. Such inhibitors could hold promise in preventing the lethal spread of IBC tumor cells in patients. Furthermore, our work identified IL-6, IL-8, and IL-10 as sufficient key inputs for macrophage-induced IBC migration. Antibody therapy directed at these microenvironmental interleukins may disrupt this signaling and prevent IBC stimulation precluding migration and metastasis.

## Methods

### Cell culture and reagents

SUM149 and SUM190 cells were maintained in Ham’s F-12 w/L-glutamine (Fisher Scientific) containing 0.5 μg/mL fungizone, 5 μg/mL gentamicin, 100 units/mL penicillin, and 100 μg/mL streptomycin (Invitrogen). Additionally, SUM149 cells were supplemented with 5% fetal bovine serum (FBS), 5 μg/mL insulin, and 1 μg/mL hydrocortisone (Sigma-Aldrich). SUM190 cells were supplemented with 0.1% bovine serum albumin, 5 μg/mL insulin, and 1 μg/mL hydrocortisone (Sigma-Aldrich). U937 and MDA-MB-231 cells were cultured in RPMI containing 10% FBS, 0.5 μg/mL fungizone, 5 μg/mL gentamicin, 100 units/mL penicillin, and 100 μg/mL streptomycin (Invitrogen). MCF10A cells were maintained in 50:50 DMEM:F12 media supplemented with 5% horse serum, 10 μg/mL insulin, 0.5 μg/mL hydrocortisone, 0.02 μg/mL epidermal growth factor, and 0.1 μg/mL cholera toxin (Sigma-Aldrich). SUM149 and SUM190 cells were maintained at 37 °C with 10% CO_2_ and all other cell lines at 37 °C with 5% CO_2_. Fresh 0.25% trypsin-EDTA in phosphate buffered saline (PBS) was used to re-suspend cells. Cytokines were purchased from R&D Systems and used at the following concentrations in all experiments: 600 ng/mL IL-8 (208-IL-050), 200 ng/mL TNFα (210-TA-020), 50 ng/mL CCL5 (278-RN-010/CF), 20 ng/mL IL-6 (206-IL-010), 10 ng/mL VEGF (293-VE-010), 5 ng/mL CCL2 (279-MC-010/CF), and 1 ng/mL IL-10 (217-IL-005).

### CRISPR cell line generation

SUM149 and SUM190 cell lines were transfected using the Nucleofector II system (Lonza) with pSpCas9(BB)-2A-GFP (PX458), which was a gift from Feng Zhang (Addgene plasmid # 48138), containing the target sequence AGGAAGACTATGATCGACTG against RhoC. Two days after transfection, single cells were sorted for GFP expression into 96 well plates. Following clonal expansion, genomic DNA was isolated and clones were screened for RhoC mutations using SURVEYOR reactions (IDT) with the following primer pair: Forward-CTGTCTTTGCTTCATTCTCCCT and Reverse-CCAGAGCAGTCTTAGAAGCCAT. Positive clones were sequenced to identify specific mutational events and immunoblotted for RhoC and RhoA.

### U937 differentiation and macrophage-conditioned media preparation

U937 cells were differentiated to macrophages as reported previously^33^,^34^. Briefly, 100 ng/mL of phorbol-12-myris-tate-12-acetate (PMA) (Thermo Fisher, BP685) was added to U937 cells in complete growth medium for 24 hours. Then, the differentiated U937 cells were rinsed and serum-free media (SFM) added and collected after another 24 hours. This macrophage-conditioned media was then concentrated using Amicon Ultra 3 K filters (EMD Millipore, UFC900324) by centrifugation at 3,220 × g at 4 °C for 50 minutes and subsequently re-diluted with fresh SFM. Expression of a panel of representative M1 and M2 genes in the PMA-differentiated macrophages as determined by qRT-PCR is shown in Supplementary Fig. S3C.

### Transwell migration assay

Corning Costar Transwell supports (Corning, 3422) were used according the manufacturer’s protocol. After trypsinization and counting, cells were aliquoted and resuspended in the appropriate media for the top insert and plated at 25,000 cells per insert. After incubation at 37 °C in either 5% or 10% CO_2_ for 24 hours, the inserts were removed and the non-migrating cells wiped away with a cotton swab. Then the migrated cells on the underside of the insert were fixed and stained with crystal violet. Images were taken at 2X of the entire migration area and the area of purple color was extracted from each image and used as a surrogate for cell number. Data represent 3 separate experiments with duplicate technical replicates for each stimulation condition.

### Microfluidic device fabrication and assembly

The migration devices were formed from a glass slide and a layer of PDMS (polydimethlysiloxane), which was fabricated on a silicon substrate by standard soft lithography. The migration channel width was 20 μm and height was 7.5 μm. The PDMS layer was bonded to the glass slide after activation by oxygen plasma treatment (80 Watts, 60 seconds) to form a complete fluidic channel. Before cell loading, collagen I (BD Biosciences, 354236) solution (1.45 mL collagen, 0.1 mL acetic acid in 50 mL deionized water) was flowed through the device for 18–24 hours in a tissue culture incubator to coat collagen on the substrate to enhance cell adhesion. Devices were then rinsed with HBSS under flow for approximately one hour to remove the residual collagen solution before use.

### Microfluidic migration assay

After rinsing the collagen coating, 100 μL of a 400,000 cells per mL cell suspension was pipetted into the top left reservoir and the loaded cells allowed to flow down the left vertical channel and align at the entrances to the horizontal migration channels. Residual cells were vacuumed and then rinsed away from the top left reservoir and complete culture medium added at the same volume to all four reservoirs in a “no flow” condition for 6 hours to allow the cells to adhere to the migration device. After cell seeding, the complete media was removed and serum-free media flowed over the attached cells for approximately one hour to flush any residual serum. Then, the top left and right inlet reservoirs were changed to the appropriate media conditions and 0 hour images captured. The device was placed in a tissue culture incubator for 24 hours after which the final migration images captured. Cell migration distance was calculated as the difference in horizontal position between the 0 hour and 24 hour images (Supplementary Fig. S2B,C). Plotted data represent 3–6 separate experiments with 1–5 technical device replicates per experiment with 16–20 cells analyzed per device for an average of 214 individual cell migration distances analyzed per plotted condition (range: 156–395 cells).

### Measurement of cytokines in conditioned media

The Bioplex Pro Human Cytokine 27-plex assay (Biorad, M500KCAF0Y) was used to measure cytokine concentrations in the specified media per the manufacturer’s protocol. Conditioned media samples were prepared fresh and diluted 1:4 in SFM before analysis. Washing was carried out utilizing a handheld magnetic plate holder and plates were read on a Bioplex MAGPIX (Biorad) machine. Internal validation of the MCM across 22 biological replicates revealed an average percent coefficient of variation of 25% for the cytokine concentrations (data not shown). Data plotted represent 2 biological replicates each with 2 technical replicates read on the same plate with the same standard curve.

### Immunoblotting

Cells were harvested in RIPA buffer (Thermo Scientific) with protease and phosphatase inhibitors (Roche Diagnostics). Immunoblotting was done after sodium dodecyl sulfate–polyacrylamide gel electrophoresis on gradient 4–15% gels (Biorad) at 30 μg protein and transferred to polyvinylidene fluoride membranes. All antibodies besides the secondary horseradish peroxidase-conjugated antibody (Santa Cruz Biotechnology) were purchased from Cell Signaling Technologies: phospho-p44/42 MAPK (ERK1/2) (Thr202/Tyr204), p44/42 MAPK (ERK1/2), phospho-p38 (Thr180/Tyr182), p38, phospho- MEK (Ser 217/221), MEK, RhoC, RhoA, ß-actin. SuperSignal West Pico Luminol/Enhancer Solution was purchased from Thermo Scientific.

### Statistical analysis

The non-parametric Kruskal-Wallis H test using Dunn’s post-hoc comparison with the Sidak correction for multiple comparisons or the non-parametric one-tailed Mann-Whitney U test were used to test for differences in the relative migration distances with a significance level set at 0.05. Pearson’s chi-squared test was used to compare proportions of non-migratory and extremely migratory cells with a significance level of 0.05 considered statistically significant. In Figures, *refers to p < 0.05, **to p < 0.01, and ***to p < 0.001 for the given test.

## Additional Information

**How to cite this article**: Allen, S. G. *et al*. Macrophages Enhance Migration in Inflammatory Breast Cancer Cells via RhoC GTPase Signaling. *Sci. Rep.* **6**, 39190; doi: 10.1038/srep39190 (2016).

**Publisher's note:** Springer Nature remains neutral with regard to jurisdictional claims in published maps and institutional affiliations.

## Supplementary Material

Supplementary Information
