# Supplementary material for: Macrophages Enhance Migration in Inflammatory Breast Cancer Cells via RhoC GTPase Signaling
Source: Sci Rep. 2016 Dec 19;6:39190. doi: 10.1038/srep39190 (PMC5171813; doi:10.1038/srep39190)
Supplement: Supplementary Information [file srep39190-s1.doc]

**Macrophages Enhance Migration in Inflammatory Breast Cancer Cells via RhoC GTPase Signaling**

Steven G. Allen*,1,2,3, Yu-Chih Chen*,4,5, Julie M. Madden3,6, Chelsea L. Fournier3, Megan A. Altemus3,7, Ayse B. Hiziroglu8, Yu-Heng Cheng4, Zhi Fen Wu3, Liwei Bao3, Joel A. Yates3, Euisik Yoon4,8,#, and Sofia D. Merajver1,3,5,7,#

1Program in Cellular and Molecular Biology, University of Michigan Medical School, Ann Arbor, MI, 48109

2Medical Scientist Training Program, University of Michigan Medical School, Ann Arbor, MI, 48109

3Department of Internal Medicine, University of Michigan, Ann Arbor, MI, 48109

4Department of Electrical Engineering and Computer Science, University of Michigan, Ann Arbor, MI 48109

5­University of Michigan Comprehensive Cancer Center, Ann Arbor, MI, 48109

6Office for Health Equity and Inclusion, University of Michigan, Ann Arbor, MI, 48109

7Program in Cancer Biology, University of Michigan Medical School, Ann Arbor, MI 48109

8Department of Biomedical Engineering, University of Michigan, Ann Arbor, MI 48109

*,# denotes equal contribution

**
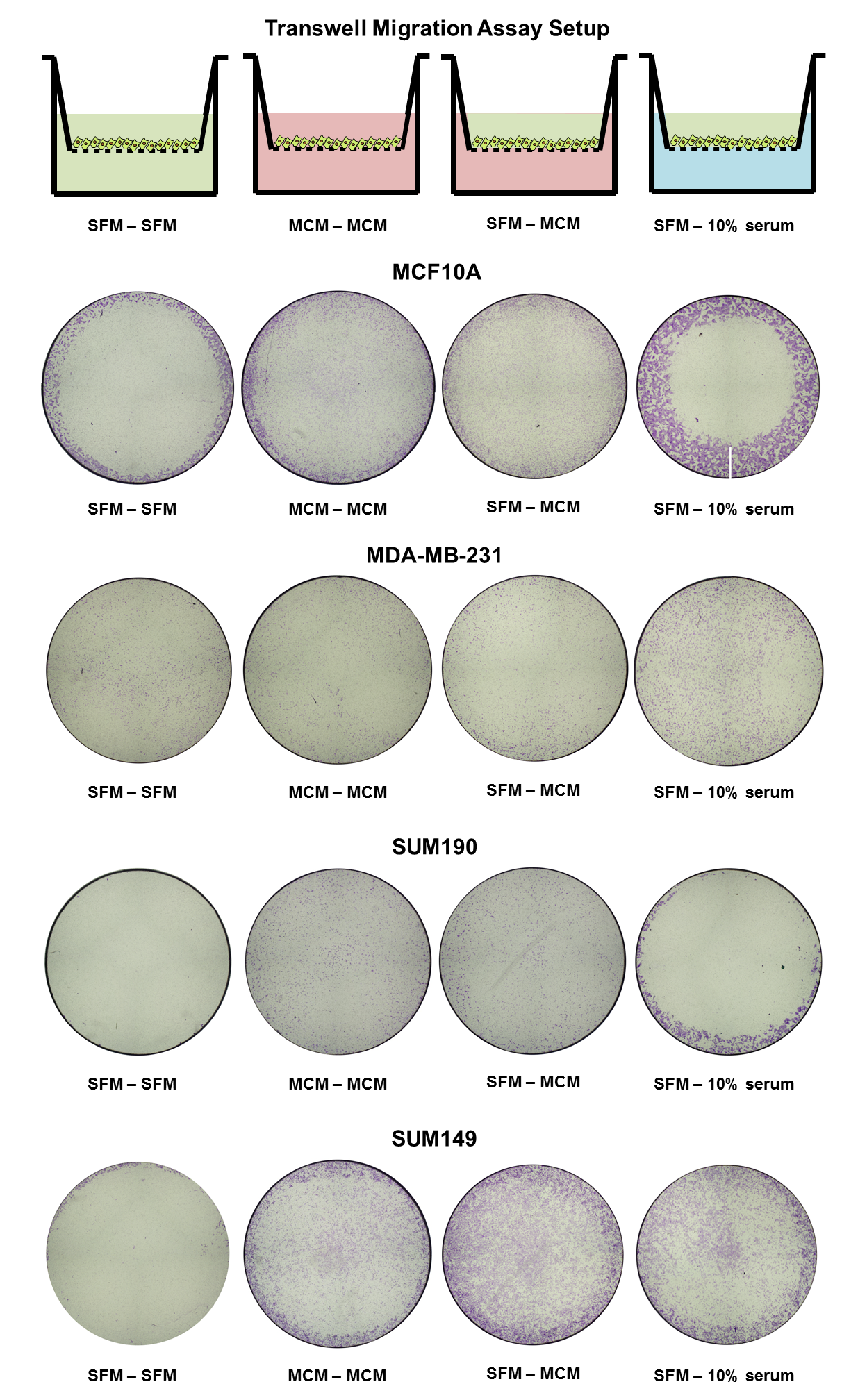
**

**Supplementary Figure S1. MCF10A, MDA-MB-231, SUM190, and SUM149 transwell migration to macrophage-conditioned media.** Schematic of transwell experimental setup and representative images of transwell membranes used to calculate percent migration for Figure 1. Cells were stained with crystal violet and the area of purple color extracted from each image and used as a surrogate for cell number. The area of each cell line’s SFM – 10% serum condition was used to normalize values.

**
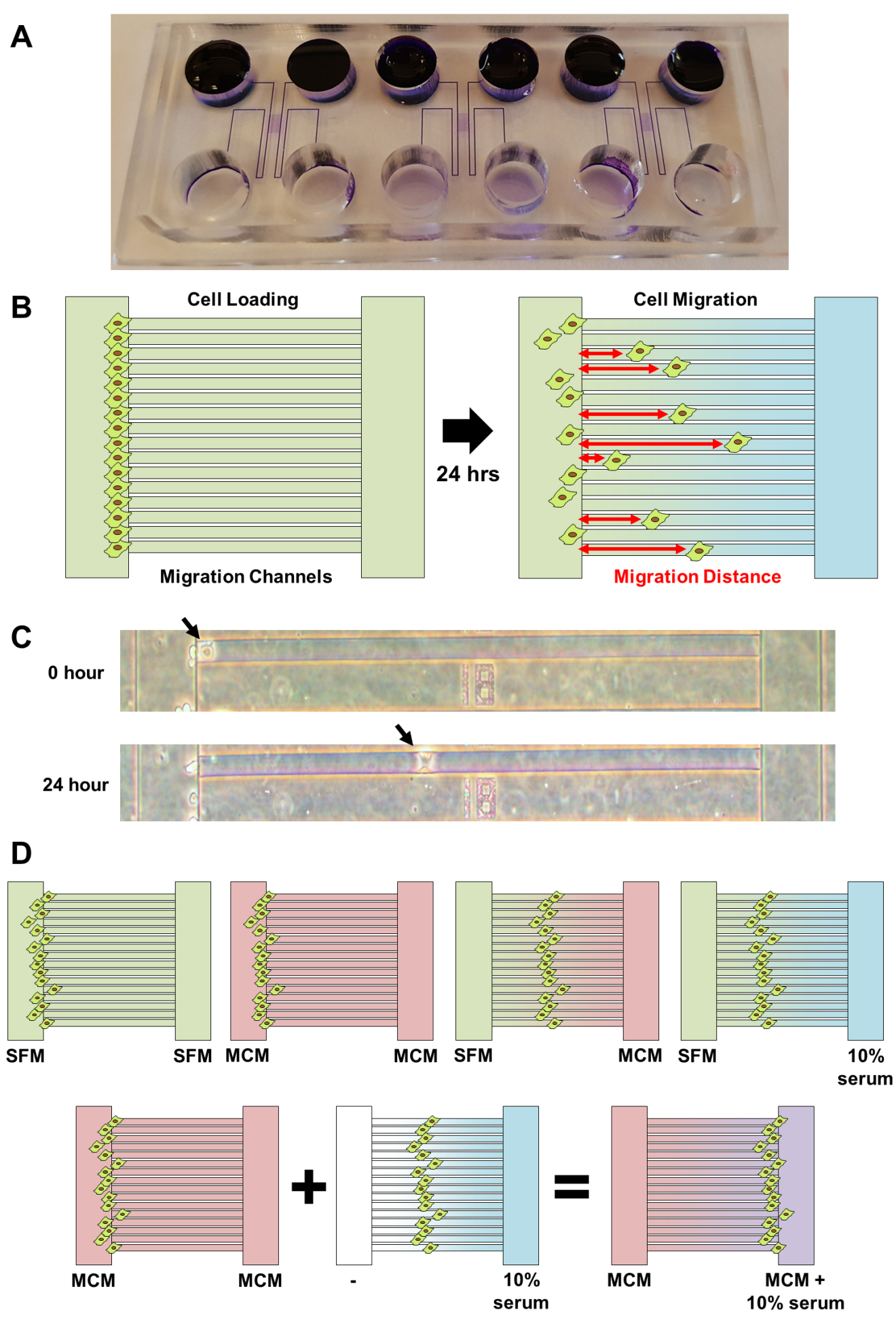
**

**Supplementary Figure S2. Schematic of the microfluidic migration device and experimental conditions.** (A) Photograph of 3 microfluidic migration devices depicting the 2 inlet (top) and 2 outlet (bottom) reservoirs with dye flowing through each device. The serpentine loading channels can be seen running vertically with the horizontal migration channels running perpendicularly between them. The devices are bonded to a standard glass slide. (B) Cells were loaded along the left side of the horizontal migration channels at the 0 hour time point and the migration distance for each cell was calculated as the difference between the 0 hour and 24 hour locations. (C) Photomicrographs of one horizontal migration channel and a migrating cell (arrows) at the 0 hour and 24 hour time points. (D) Schematic of the various concentration gradients and experimental conditions tests in the microfluidic migration devices. The approximate net resultant average migration distance for each condition is also depicted.


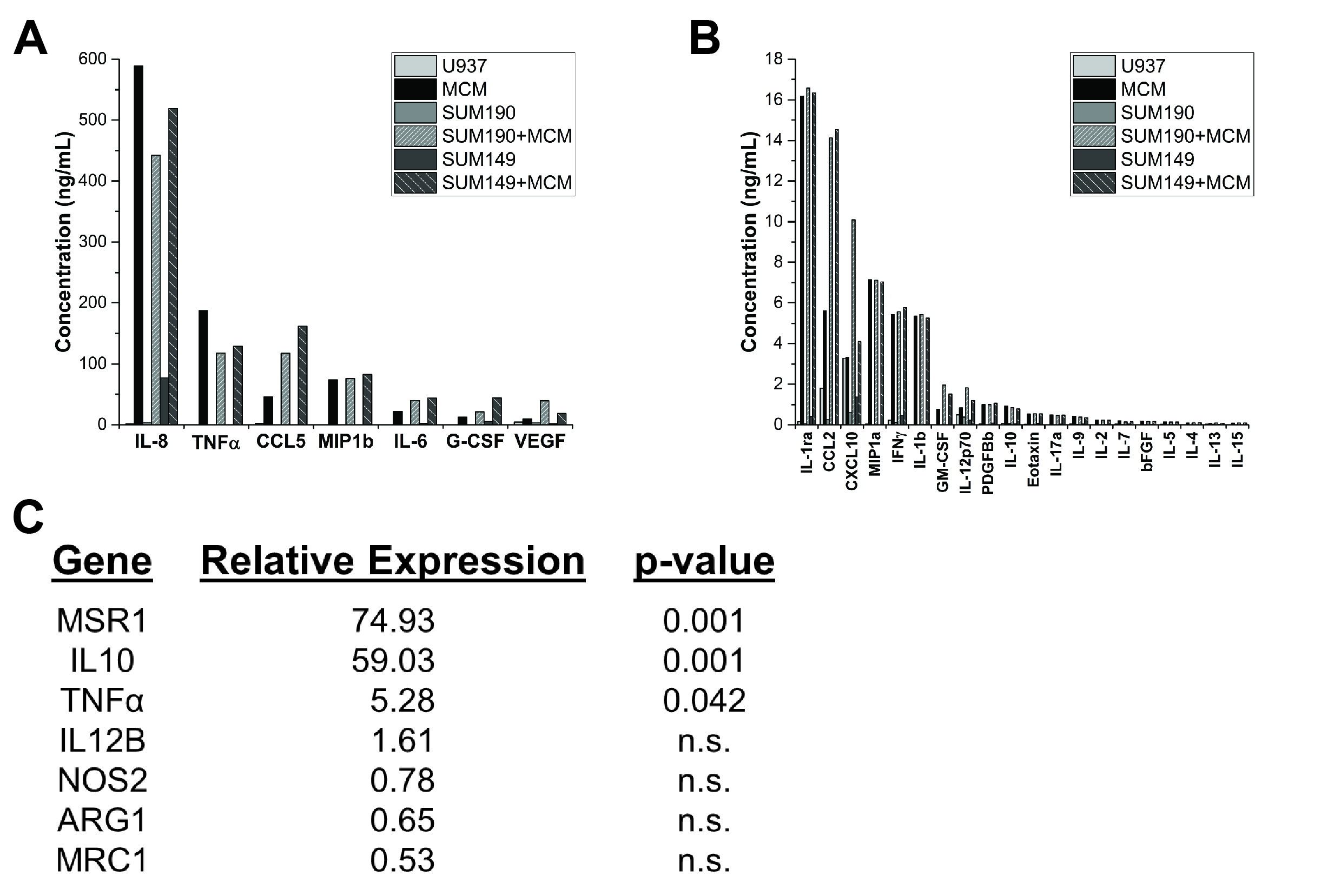
**Supplementary Figure S3. Conditioned media cytokine concentration and macrophage gene expression.** The concentration of all measured 27 cytokines in different media conditions is plotted in (A) and (B). The first column represents undifferentiated U937 monocyte conditioned media. The second column represents the PMA-differentiated U937 MCM (the media used for experimental stimulation of IBC cells). The third and fifth columns show the basal secretion of cytokines by unstimulated SUM190 and SUM149 cells, respectively. The fourth and sixth columns represent SUM190 and SUM149 cells stimulated with MCM. (C) Relative gene expression of PMA-differentiated U937 macrophages as compared to undifferentiated U937 monocytes. For the qRT-PCR data, REST 2009 analysis software was used to assess significance using three housekeeping genes (RPL22, RPL30, and GAPDH) for normalization and with 5,000 iterations.


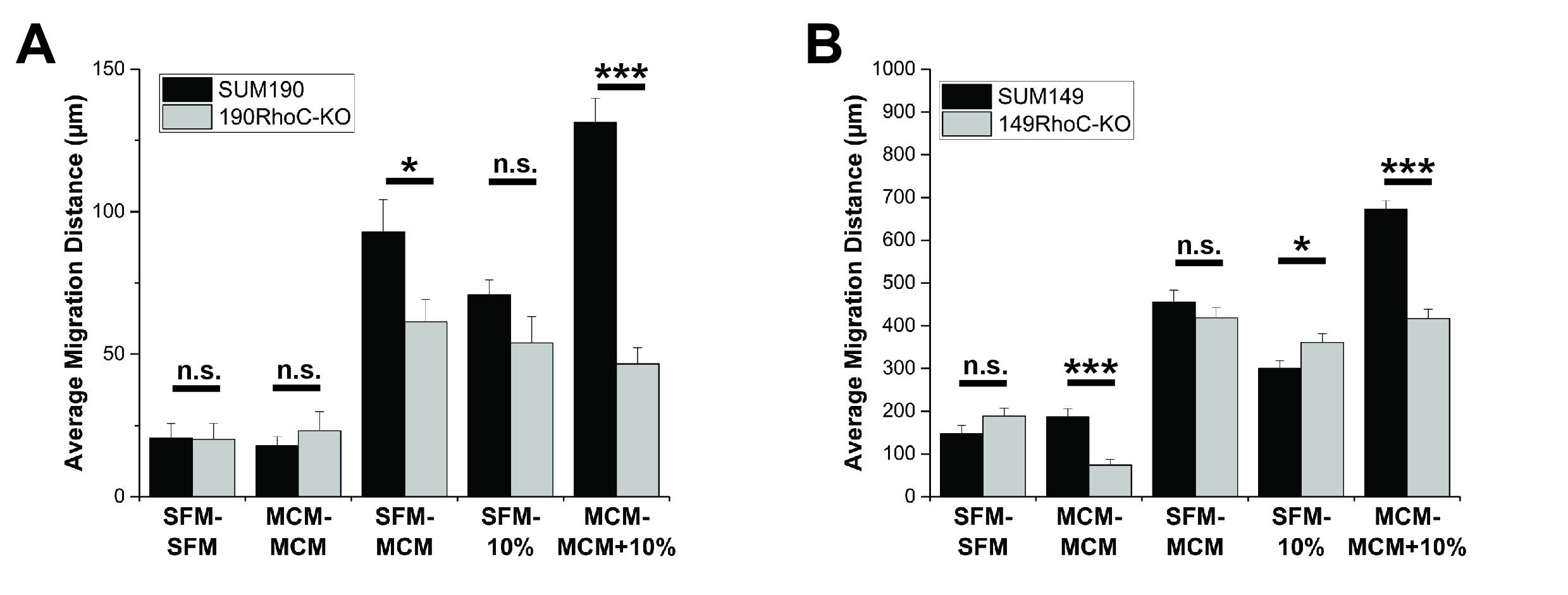
**Supplementary Figure S4. SUM190, 190RhoC-KO, SUM149, and 149RhoC-KO average microfluidic migration.** Average microfluidic migration distances for (A) SUM190 and 190RhoC-KO and (B) SUM149 and 149RhoC-KO cells. (A) SUM190 and 190RhoC-KO cells did not migrate differently to SFM – 10% serum control, but the absence of RhoC in 190RhoC-KO cells completely abrogated the enhanced migration response to MCM – MCM+10% serum. (B) 149RhoC-KO cells migrated further in response to the SFM – 10% serum condition than SUM149 cells. This would have decreased the likelihood of finding a difference in the extreme migration condition; however, the absence of RhoC still completely abrogated the enhanced migration effect in 149RhoC-KO cells. * denotes p < 0.05, *** denotes p < 0.001, two-tailed Student’s t-test, error bars represent s.e.m.
